# Eco-friendly synthesis of an antimicrobial polymer (1,4-bis(methacryloyl)piperazine) via maghnite catalysis: *in vitro* activity and *in silico* drug-likeness

**DOI:** 10.3389/fchem.2026.1800761

**Published:** 2026-04-29

**Authors:** Samira Derkaoui, Farouk Boudou, Ahcene Keziz, Alaeddine Berkane, Huda Alsaeedi, David Cornu, Mikhael Bechelany, Ahmed Barhoum

**Affiliations:** 1 Department of Chemistry, Faculty of Exact and Applied Sciences, Laboratory of Polymer Chemistry, University ORAN 1 Ahmed Ben Bella, Oran, Algeria; 2 Department of Physical Science and Technology, Higher Normal School of Saida, Saïda, Algeria; 3 Department of Applied Molecular Genetics, Faculty of Natural and Life Sciences, University of Science and Technology of Oran, Oran, Algeria; 4 Department of Physics, Physics and Chemistry of Materials Laboratory, University of M’sila, M’sila, Algeria; 5 Department of Chemistry, Faculty of Sciences, Laboratory of Chemistry, Synthesis, Properties, and Applications (LCSPA), Dr. Moulay Tahar University of Saida, Saïda, Algeria; 6 Department of Chemistry, College of Science, King Saud University, Riyadh, Saudi Arabia; 7 Institut Européen des Membranes, IEM, UMR 5635, Université Montpellier, CNRS, Montpellier, France; 8 School of Chemical and Biopharmaceutical Sciences, Technological University Dublin, Dublin, Ireland

**Keywords:** antimicrobial activity, drug-likeness, maghnite catalysis, molecular docking, molecular dynamics, NBMP, piperazine polymer, toxicity predictions

## Abstract

**Background:**

Poly (1,4-bis(methacryloyl)piperazine) (poly (NBMP)) is a piperazine-based polymer with potential biomedical applications. Green clay catalysts, maghnite-H^+^ and maghnite-Na^+^; offer an eco-friendly approach for monomer (NBMP) and polymer (poly (NBMP)) synthesis with improved yields and low toxicity.

**Aim:**

To synthesize poly (NBMP) via green catalysis, evaluate its structural properties, and investigate its antimicrobial potential along with drug-likeness and molecular interaction profiles of its monomer.

**Method:**

The monomer and polymer were synthesized using varying amounts of maghnite-H^+^ and maghnite-Na^+^ at controlled temperatures. Structural characterization was performed using FTIR, ^1^H and ^13^C NMR, SEM, and DSC. Antibacterial activity was tested against Gram-positive (*S. aureus, L. monocytogenes*) and Gram-negative bacteria (*E. coli, P. aeruginosa, K. pneumoniae*). Drug-likeness, toxicity predictions, molecular docking, and molecular dynamics (MD) simulations were conducted to assess binding affinities and complex stability of NBMP with the target bacterial proteins.

**Results:**

Monomer yield increased from 40% to 72% with 0–10 wt% maghnite-H^+^, while polymer yield rose from 5% to 70% using 0–15 wt% catalyst. Poly (NBMP) exhibited significant antibacterial activity, with inhibition zones of 32 μg/mL against *S. aureus* and 16 μg/mL against *E. coli.* Docking studies revealed moderate binding to *K. pneumoniae* FabG (PDB ID: 6T77, −6.1 kcal/mol). MD simulations confirmed stable complexes with RMSD values of 0.43 nm for *E. coli* DNA gyrase (PDB ID: 1KZN) and 0.19 nm for *K. pneumoniae* FabG, along with low RMSF and compact radius of gyration (0.04–0.07 nm).

**Discussion:**

The findings demonstrate that NBMP forms stable interactions with bacterial proteins, supporting its broad-spectrum antimicrobial activity. The eco-friendly synthesis, favorable drug-likeness, and structural stability highlight NBMP as a promising candidate for future biomedical applications. Further *in vitro* and *in vivo* studies are recommended to validate its therapeutic potential.

## 1 Introduction

Polymers are macromolecules composed of repeating structural units that can be engineered to obtain specific physical, chemical, and biological properties. This versatility allows their use in various forms, such as fibers, films, and coatings, which are particularly suitable for carrying therapeutic agents in drug delivery systems (Arash et al., 2024). In biomedical applications, polymers are highly valued because they can be tailored with specific functionalities, such as biocompatibility, biodegradability, and antimicrobial activity that are essential for effective drug delivery vehicles (Alkarri et al., 2024; Anaif et al., 2024). Polymers derived from natural and synthetic sources exhibit excellent compatibility with biological systems and are widely used in tissue engineering and regenerative medicine due to their low toxicity and functional adaptability (Harini et al., 2025). Synthetic polymers, including poly (ethylene glycol) and poly (lactic acid), are widely employed in drug delivery systems and tissue engineering owing to their modifiability with bioactive compounds, which enhances their therapeutic performance (Jie et al., 2024). Antimicrobial resistance has emerged as a critical global health issue, necessitating the development of alternative therapeutic strategies beyond conventional antibiotics (Majumder et al., 2020). In this context, antimicrobial polymers have gained significant attention due to their ability to mimic natural antimicrobial peptides and exhibit strong activity against a broad spectrum of microorganisms, including multidrug-resistant strains. These polymers act through multiple mechanisms, such as disruption of microbial membranes, induction of oxidative stress, and interference with intracellular processes, thereby reducing the likelihood of resistance development and enhancing therapeutic efficacy (Harini et al., 2024). Their multifunctional nature and structural tunability make them promising candidates for next-generation antimicrobial agents and biomedical applications. The antibacterial mechanism of polymeric systems is mainly driven by electrostatic interactions between positively charged polymers and negatively charged bacterial membranes, leading to membrane disruption and increased permeability (Qiu et al., 2020). Some polymers can also penetrate cells and interfere with intracellular components such as proteins and nucleic acids, while the generation of reactive oxygen species (ROS) induces oxidative damage to essential cellular structures (Jankoski et al., 2025). This multimodal action enhances antibacterial efficacy and reduces the risk of resistance development.

To meet drug-likeness requirements, polymers must exhibit favorable characteristics such as appropriate molecular weight, adequate solubility, and balanced lipophilicity to ensure efficient absorption and distribution within biological systems (Bruno et al., 2021). Low toxicity and minimal adverse effects are also critical for clinical applicability (Breijyeh and Karaman, 2023). In this regard, polymer-based drug delivery systems have demonstrated the ability to enhance pharmacokinetic properties, enable controlled and sustained release, and improve therapeutic targeting, thereby minimizing side effects and increasing treatment efficiency (Ding et al., 2024). Advanced computational modeling tools, including SwissADME for pharmacokinetic and drug-likeness predictions, AutoDock Vina for binding affinity estimation, and molecular dynamics packages such as GROMACS and AMBER, enable the optimization of polymer structures to improve their interactions with biological targets (Kawashima et al., 2023). Through controlled synthesis strategies, polymer architectures can be fine-tuned to enhance bioactivity, improve therapeutic outcomes, and minimize side effects, thereby advancing the development of efficient drug delivery systems that meet modern medical demands.

In silico drug-likeness analysis provides valuable insights into the potential efficacy and safety of polymers intended for drug delivery applications. This computational approach relies on predictive models and algorithms, such as SwissADME and ADMETlab, to evaluate key polymer properties, including molecular weight, hydrophilicity, lipophilicity, solubility, permeability, and structural complexity (Daina et al., 2017). These platforms facilitate the assessment of parameters influencing absorption, distribution, metabolism, and excretion (ADME) (Hiroshi et al., 2023). For example, SwissADME predicts molecular descriptors such as topological polar surface area, which is critical for estimating a polymer’s ability to permeate cell membranes (Landis et al., 2017). Additionally, pkCSM and toxicity estimation tools provide toxicological predictions by identifying potential adverse interactions between polymers and biological systems. Recent advances also highlight the structural tunability of polymers, enabling the design of sophisticated delivery platforms, including gene delivery systems and nanoscale carriers, which enhance therapeutic precision and efficiency (Jogdeo et al., 2024). These analyses consider factors such as hydrogen bond donors and acceptors, rotatable bonds, and polymer polarity, all of which influence solubility and bioavailability (Macan, 2023). Molecular docking tools such as AutoDock Vina and Molecular Operating Environment further simulate binding affinities, allowing evaluation of polymer compatibility with target sites and refining their therapeutic potential. Overall, *in silico* techniques enable the optimization of polymer structures prior to synthesis, streamlining drug development while improving pharmacokinetic profiles and reducing toxicity (Mantravadi et al., 2019). Furthermore, recent developments in polymer-based nanoformulations have demonstrated the potential for pH-responsive drug delivery systems, which improve drug stability and enable controlled release in specific physiological environments. Such systems are particularly relevant in cancer therapy, where differential pH conditions between healthy and diseased tissues can be exploited to enhance therapeutic efficacy and reduce systemic toxicity (Agrawal et al., 2025). Poly (1,4-bis(methacryloyl)piperazine) (poly (NBMP)) demonstrates considerable versatility and promise for biomedical applications, particularly in antimicrobial therapies and drug delivery systems (Derkowska-Zielinska et al., 2021; Manohara et al., 2021). This polymer can be synthesized using various catalytic polymerization techniques, each contributing to enhanced functional properties. Free-radical polymerization, typically initiated by azobisisobutyronitrile, generates reactive sites for monomer propagation, while anionic polymerization using sodium naphthalene or potassium tert-butoxide enables precise control over molecular weight and polydispersity. Coordination–insertion polymerization employing catalysts such as titanium (IV) isopropoxide or zirconium-based complexes allows the incorporation of specific functional groups, thereby enhancing the antimicrobial activity of poly (NBMP). Furthermore, organocatalysts, including proline and bifunctional amines, facilitate polymerization without metal catalysts, an advantage for applications requiring high biocompatibility. Lewis acids, such as aluminum chloride and tin (IV) chloride, also act as initiators that modify polymer architecture, improving interactions with microbial membranes and contributing to antimicrobial efficacy (Vogel et al., 2016). Owing to its tunable structure and antibacterial potential, poly (NBMP) has been investigated for use in medical coatings, drug delivery systems, and tissue engineering scaffolds, where controlled polymerization techniques allow customization for specific therapeutic needs (Shaquiquzzaman et al., 2015; Shundrina et al., 2015).

The aim of the present work was to synthesize poly (NBMP) via catalytic polymerization using environmentally friendly maghnite-H^+^ and maghnite-Na^+^ as green catalysts (Derkaoui et al., 2019). The study further evaluated the antimicrobial activity of the synthesized polymer, along with its drug-likeness, through molecular docking and molecular dynamics simulations. Antimicrobial activity was assessed against clinically relevant bacterial strains, including *Escherichia coli (ATCC 25922), Pseudomonas aeruginosa* (ATCC 27853), and *Klebsiella pneumoniae* (ATCC 27853) as Gram-negative bacteria, and *Staphylococcus aureus* (ATCC 25923) and *Listeria monocytogenes* (ATCC 15313) as Gram-positive bacteria. The significance of this study lies in the development of poly (NBMP) as an innovative biomedical material combining effective drug delivery potential with inherent antimicrobial properties. Maghnite-H^+^ and maghnite-Na^+^, derived from natural clay minerals, exhibit good biocompatibility, supporting their safe use in biomedical applications. Their solid and heterogeneous nature allows easy recovery and reuse, making the polymerization process cost-effective, environmentally friendly, and sustainable. Additionally, these catalysts can serve a dual function as reinforcing fillers, improving the mechanical and functional properties of the resulting polymer while maintaining high yields and product purity. Although maghnite-H^+^ and maghnite-Na^+^ display minimal intrinsic antimicrobial activity, their effects are mainly attributed to microbial membrane disruption through metal ion release and the generation of reactive oxygen species (Medina et al., 2020; Wang et al., 2020; Wang et al., 2021).

## 2 Experimental

### 2.1 Materials

Chemicals and reagents were obtained from reliable commercial sources and used without further purification. Methacrylic anhydride (C_8_H_10_O_3_) and dichloromethane (CH_2_Cl_2_) were purchased from Sigma-Aldrich (Algeria). Piperazine (C_4_H_10_N_2_) was from Acros Organics. Magnesium sulfate (MgSO_4_) and sodium chloride (NaCl) were supplied by Biochem (France). Sulfuric acid (H_2_SO_4_) and barium nitrate (Ba(NO_3_)_2_) were from Sigma-Aldrich. Silver nitrate (AgNO_3_) was purchased from Loba Chemie (India). Raw maghnite, a natural montmorillonite clay, was from ENOF Company (Maghnia, Algeria).

### 2.2 Preparation of maghnite catalysts

**FIGURE 1:**
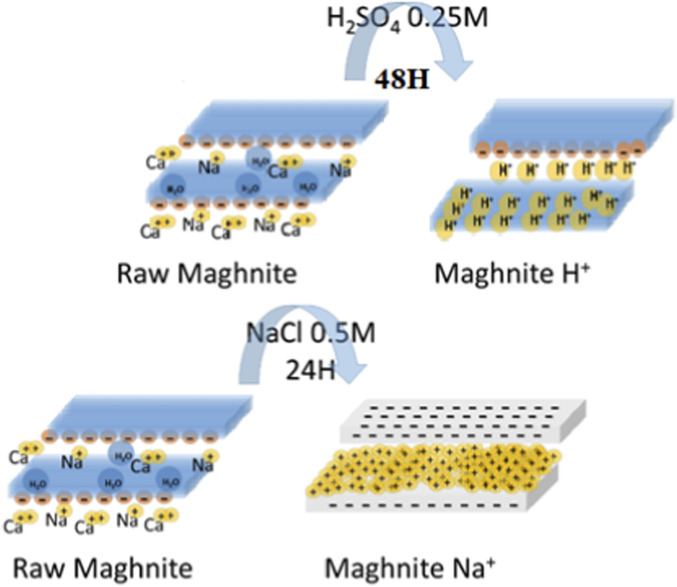
Schematic representation of the preparation of maghnite-H^+^ and maghnite-Na^+^ catalysts.

The maghnite-H^+^ and maghnite-Na^+^ catalysts were prepared according to the procedure reported by Belbachir and Bensaoula (2001), Belbachir and Bensaoula (2006) to provide acidic (H^+^) and basic (Na^+^) environments suitable for anionic addition polymerization and other reaction steps (see Figure 1). For the preparation of maghnite-H^+^, 20 g of raw maghnite was first milled using a ceramic ball mill for 20 min to reduce particle size and increase surface area. The milled material was then dried in an oven at 105 °C for 2 h to remove residual moisture. The dried maghnite was transferred to a 1 L Erlenmeyer flask containing 500 mL of distilled water. Under continuous stirring with a magnetic stirrer, 0.25 M sulfuric acid (H_2_SO_4_) was gradually added. The suspension was stirred at room temperature for 48 h to ensure complete saturation of maghnite with hydrogen ions (H^+^). After treatment, maghnite-H^+^ was separated by filtration and washed repeatedly with distilled water to eliminate residual sulfate ions. The presence of sulfate ions in the washings was checked using barium nitrate (Ba(NO_3_)_2_); the absence of a white precipitate confirmed their complete removal. The purified maghnite-H^+^ was finally dried at 105 °C and stored in a desiccator. Maghnite-Na^+^ was prepared using a similar ion-exchange procedure. Briefly, 20 g of raw maghnite was dispersed in 500 mL of 1 M sodium chloride (NaCl) solution in an Erlenmeyer flask and stirred at room temperature for 24 h to promote complete exchange between sodium ions (Na^+^) and the native cations present in the maghnite structure. The resulting maghnite-Na^+^ was then recovered by filtration and thoroughly washed with distilled water to remove excess chloride ions. The washing process was monitored using silver nitrate (AgNO_3_); the absence of a white precipitate indicated complete chloride removal. Finally, maghnite-Na^+^ was dried at 105 °C and stored in a desiccator.

### 2.3 Synthesis of the NBMP monomer

**FIGURE 2:**
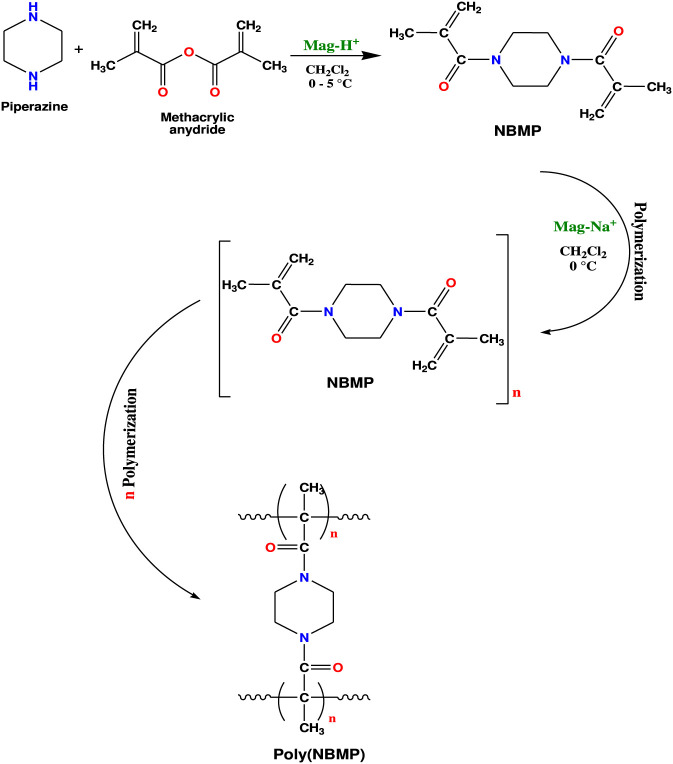
Schematic illustration of the synthesis of 1,4-bis (methacryloyl) piperazine (NBMP) monomer and its subsequent polymerization catalyzed by maghnite-H^+^ and maghnite-Na^+^, respectively.

For monomer synthesis, 0.86 g of piperazine was mixed with varying amounts of maghnite-H^+^ catalyst (3–20 wt%; see Table 1) and stirred at room temperature for 30 min. Subsequently, 0.2 mol (30 mL) of methacrylic anhydride was added dropwise at a molar ratio of 2:1 (methacrylic anhydride to piperazine). The reaction mixture was cooled to 5 °C and maintained at this temperature for 2 h to control the exothermic reaction (Figure 2). After completion, the catalyst was recovered by filtration. The filtrate was transferred to a separatory funnel and washed with 5% NaOH solution to neutralize any unreacted methacrylic anhydride. The organic phase was then extracted three times with 30 mL of dichloromethane. The combined organic extracts were dried over anhydrous MgSO_4_, and the solvent was removed under reduced pressure. The resulting crude product was recrystallized from cold diethyl ether to obtain pure 1,4-bis(methacryloyl)piperazine as a white crystalline solid.

**TABLE 1:** Effect of maghnite-H^+^ and maghnite-Na^+^ catalyst loading on the synthesis yields of NBMP monomer and poly (NBMP) polymer.

Samples	Synthesis conditions	Catalyst (wt%)	Yield (%)
NBMP monomer	Piperazine (0.86 g) + methacrylic anhydride (0.2 mol); catalyzed by maghnite-H^+^; stirred at room temp for 30 min, cooled at 5 °C for 2 h; catalyst filtered off	3	40
		5	60
		10	72
		15	72
		20	72
Poly(NBMP)	NBMP (1 g) polymerized in dichloromethane at 0 °C under N_2_ for 24 h; catalyzed by maghnite-Na^+^; catalyst filtered off	3	5
		5	30
		10	59
		15	70
		20	70

### 2.4 Synthesis of poly (NBMP)

The synthesized NBMP monomer was polymerized using maghnite-Na^+^ as catalyst at varying loadings (3–20 wt%; see Table 1). In sealed reaction tubes, 1 g of NBMP was dissolved in dichloromethane and mixed with the appropriate amount of maghnite-Na^+^ catalyst. The reaction mixture was cooled to 0 °C using an ice bath and stirred under a dry nitrogen atmosphere to prevent moisture and oxygen interference. After 24 h of polymerization, the reaction mixture was filtered to recover the maghnite-Na^+^ catalyst. The polymer-containing filtrate was then precipitated by pouring it into cold methanol. The precipitated polymer was collected by filtration, washed several times with methanol to remove residual impurities, and dried under vacuum at 40 °C. The final polymer was weighed and stored for further characterization.

### 2.5 Physicochemical characterization

The maghnite-H^+^ and maghnite-Na^+^ catalysts were characterized by X-ray diffraction (XRD) using a D8 Advance Bruker AXS diffractometer (Germany). The synthesized polymer was characterized under controlled conditions using several complementary analytical techniques. Fourier Transform Infrared (FTIR) spectroscopy was performed with a Bruker Alpha-P ATR spectrometer (No. 9501165, France) over the spectral range of 400–4,000 cm^-1^ to identify the functional groups present in the polymer. For analysis, the polymer was mixed with KBr and pressed into pellets. Spectra were recorded at room temperature with a resolution of 4 cm^-1^ and averaged over 32 scans to improve the signal-to-noise ratio. ^1^H and ^13^C Nuclear Magnetic Resonance (NMR) spectroscopy was carried out using a Bruker 300 MHz NMR spectrometer (Germany) to confirm the polymer structure. Tetramethylsilane (TMS) was used as the internal standard, and deuterated chloroform (CDCl_3_) served as the solvent. All spectra were recorded at room temperature. Differential Scanning Calorimetry (DSC) analysis was conducted using a PerkinElmer STA 6000 thermal analyzer (United States) to evaluate the thermal stability of the polymer. Measurements were performed under a nitrogen atmosphere at a heating rate of 10 °C·min^-1^, from room temperature up to 600 °C. The surface morphology of the polymer was examined by Scanning Electron Microscopy (SEM) using a JEOL JSM-F100 microscope (Japan). Prior to analysis, samples were coated with a thin gold layer to enhance electrical conductivity. SEM observations were performed at an accelerating voltage of 15 kV at the University of Haute Alsace, Mulhouse, France.

### 2.6 Antibacterial activity

The antibacterial activity of poly (NBMP) was evaluated against selected Gram-positive and Gram-negative bacterial strains. The tested microorganisms included *Escherichia coli* ATCC 25922, *Pseudomonas aeruginosa* ATCC 27853, and *Klebsiella pneumoniae* ATCC 27853 (Gram-negative), as well as *Staphylococcus aureus* ATCC 25923 and *Listeria monocytogenes* ATCC 15313 (Gram-positive). All strains were cultured for 24 h prior to testing. Bacterial cells were harvested and suspended in sterile saline solution to obtain a concentration of approximately 1 × 10^8^ CFU·mL^-1^, adjusted according to the 0.5 McFarland standard. For antibacterial assessment, 20 mL of sterile Mueller–Hinton agar (containing 2.0% beef extract, 17.5% casein acid hydrolysate, 1.5% starch, and 1.5% agar) was poured into sterile Petri dishes and allowed to solidify. The surface of each agar plate was uniformly inoculated using a sterile cotton swab dipped into the bacterial suspension. Plates were then left to dry for 15–20 min to allow bacterial adherence.

Wells with a diameter of 6.0 mm were aseptically punched into the agar and filled with 50 µL of methanolic solutions containing different concentrations of poly (NBMP), namely 5,000, 2,500, 1,250, 630, and 312 μg·mL^-1^. In parallel, standard antibiotic discs were used as positive controls, including gentamicin (50 µg/disc), cefazolin (20 µg/disc), pipemidic acid (30 µg/disc), and colistin (10 µg/disc). All discs had a diameter of 6.0 mm and were placed on the agar surface alongside the polymer samples. Poly (NBMP) samples and antibiotic discs were carefully positioned to avoid overlapping inhibition zones.

The inoculated plates were incubated at 37 °C for 24 h. After incubation, the diameters of the inhibition zones were measured in millimeters using a caliper. All experiments were performed in triplicate, and the results were expressed as mean ± standard deviation (SD). Statistical analysis was carried out using SigmaPlot for Windows (version 11.0). Differences between groups were assessed by one-way analysis of variance (ANOVA), followed by Tukey’s *post hoc* test.

### 2.7 Drug-likeness and toxicity predictions

The SwissADME platform (http://www.swissadme.ch) was used to evaluate the drug-likeness and predicted pharmacokinetic properties of the monomeric unit of poly (NBMP). SwissADME provides a comprehensive set of computational tools for calculating key physicochemical descriptors relevant to drug discovery. These include predictions related to absorption, distribution, metabolism, and excretion (ADME), as well as pharmacokinetic parameters and drug-likeness indices such as Lipinski’s Rule of Five (Lipinski, 2001), which is commonly used to estimate oral bioavailability potential. The chemical structure of NBMP was uploaded to the SwissADME server in a standard format (e.g., SMILES or InChI). The platform calculated several physicochemical properties, including molecular weight, lipophilicity (logP), the number of hydrogen bond donors and acceptors, and topological polar surface area. These parameters provide insight into the compound’s absorption behavior, bioavailability, and potential toxicity. In addition, SwissADME applies medicinal chemistry filters, including synthetic accessibility and drug-likeness alerts, to evaluate the feasibility of developing the compound as a therapeutic agent. Toxicity predictions were performed using the eMolTox web-based tool, which is designed to identify potential toxic liabilities associated with chemical structures. This platform analyzes the chemical composition of the compound to predict its safety profile and potential adverse effects. Several toxicity endpoints were evaluated, including hepatotoxicity, cardiotoxicity, and cytotoxicity. These predictions are essential for assessing the overall safety of the compound and its suitability for further development, particularly in cases where promising therapeutic properties are observed.

### 2.8 Molecular docking analysis

The synthesized monomeric unit of poly (NBMP) ligand was designed using MarvinSketch (Version 5.10.0) and saved in the Protein Data Bank (PDB) format. The PDB file was then processed with AutoDock Tools to generate the corresponding PDBQT file, suitable for molecular docking with AutoDock Vina. The crystal structures of the following target proteins were retrieved from the RCSB Protein Data Bank (www.rcsb.org): *Escherichia coli* DNA gyrase (PDB ID: 1KZN), *Staphylococcus aureus* penicillin-binding proteins (PDB IDs: 3HUN, 7KCY, 5OJ0, 6R3X), *Listeria* monocytogenes listeriolysin O (PDB ID: 4CDB), *Klebsiella pneumoniae* NADPH-dependent FabG (PDB ID: 6T77), and *Pseudomonas aeruginosa* quorum-sensing receptor RhlR (PDB ID: 8DQ1). Prior to docking, all water molecules were removed from the protein structures, and polar hydrogen atoms were added using AutoDock Tools to ensure accurate docking simulations. Docking calculations were carried out with AutoDock Vina integrated into the PyRx Virtual Screening Tool v.8. The binding sites were defined by adjusting the grid boxes around the active pockets of each protein using the AutoGrid feature in PyRx. The grid box dimensions and center coordinates for each target were as follows:

- DNA gyrase (PDB ID: 1KZN): 46.49 Å × 46.89 Å × 41.22 Å, centered at (19.150, 30.393, 34.745 Å).
- Penicillin-binding protein (PDB ID: 3HUN): 63.72 Å × 65.04 Å × 97.59 Å, centered at (−33.648, 13.630, −10.162 Å).
- Listeriolysin O (PDB ID: 4CDB): 44.51 Å × 72.13 Å × 121.33 Å, centered at (−11.691, −8.144, −14.361 Å).
- Penicillin-binding protein (PDB ID: 5OJ0): 63.72 Å × 65.04 Å × 97.59 Å, centered at (31.712, −15.361, 52.877 Å).
- Penicillin-binding protein (PDB ID: 6R3X): 63.72 Å × 65.04 Å × 97.59 Å, centered at (7.114, 40.689, 15.918 Å).
- FabG (PDB ID: 6T77): 55.67 Å × 78.10 Å × 53.10 Å, centered at (−12.659, −1.511, −13.323 Å).
- Penicillin-binding protein (PDB ID: 7KCY): 63.72 Å × 65.04 Å × 97.59 Å, centered at (−6.960, 17.986, 15.628 Å).
- Quorum-sensing receptor RhlR (PDB ID: 8DQ1): 57.05 Å × 94.89 Å × 115.85 Å, centered at (130.594, 141.381, 163.014 Å).

Docking simulations were performed on a computer running Microsoft Windows 10 Professional (Service Pack 3), equipped with an Intel(R) Core™ i3-7020U CPU @ 2.30 GHz and 4.0 GB RAM. After docking, the binding energies and ligand–protein complex conformations were analyzed using BIOVIA Discovery Studio Visualizer. Redocking of the co-crystallized ligands into their respective protein binding sites was performed using AutoDock Vina. The reliability of the docking protocol was evaluated by reporting the Vina RMSD values for the top three poses, with each protein using its own grid center and dimensions. This analysis provided detailed information on ligand binding affinities, types of interactions, and spatial arrangement within the active sites of the target proteins.

### 2.9 Molecular dynamic simulations

Molecular dynamics (MD) simulations were performed on the target proteins that exhibited the strongest binding affinities and most favorable interactions with the synthesized poly (NBMP) ligand. These proteins included *E. coli* DNA gyrase (PDB ID: 1KZN), *K. pneumoniae* NADPH-dependent FabG (PDB ID: 6T77), *S. aureus* penicillin-binding proteins (PDB IDs: 3HUN, 7KCY, 5OJ0, 6R3X), *L. monocytogenes* listeriolysin O (PDB ID: 4CDB), and *P. aeruginosa* quorum-sensing receptor RhlR (PDB ID: 8DQ1). The aim of these simulations was to evaluate the stability and dynamic behavior of the protein-ligand complexes over time. Simulations were conducted using GROMACS 2023-GPU with the CHARMM36-2019 force field. Initially, the protein-ligand complexes identified from molecular docking were prepared, ensuring proper protonation states and geometry. Each complex was then solvated in a cubic box using TIP3P water molecules, maintaining a minimum distance of 1.0 nm between the solute and the box edges to accurately replicate physiological conditions. Sodium (Na^+^) and chloride (Cl^−^) ions were added to neutralize the system and achieve an ionic strength of 0.15 M. Energy minimization was performed using the steepest descent algorithm to remove steric clashes and optimize the system geometry, with a convergence criterion set to a maximum force of 1,000 kJ/mol·nm. Equilibration was carried out in two steps: first, an NVT ensemble simulation (constant number of particles, volume, and temperature) at 300 K for 2 ns using a velocity-rescaling thermostat, followed by an NPT ensemble simulation (constant number of particles, pressure of 1 bar, and temperature) for 2 ns. Production MD simulations were then executed for a total of 250 ns at 300 K and 1 bar, using a time step of 2 fs. Trajectory files were recorded throughout the simulations for subsequent analyses. Key parameters evaluated included: Root-mean-square deviation (RMSD): to monitor the overall structural deviations of the protein backbone. Root-mean-square fluctuation (RMSF): to analyze the flexibility of individual residues. Radius of gyration (Rg): to assess the compactness of the protein-ligand complexes over time. Trajectory analyses were performed using GROMACS built-in tools, and the simulations were visualized using Visual Molecular Dynamics (VMD). This comprehensive approach provided detailed insights into the stability, flexibility, and structural dynamics of the poly (NBMP)–protein complexes. MD simulations were executed on a Linux-based workstation (Ubuntu 20.04.6) equipped with an Intel Core i9-14000K processor, 16 GB RAM, and an NVIDIA GeForce RTX 4070 Ti Super GPU.

## 3 Results and discussion

### 3.1 Characteristics of maghnite catalysts

**FIGURE 3:**
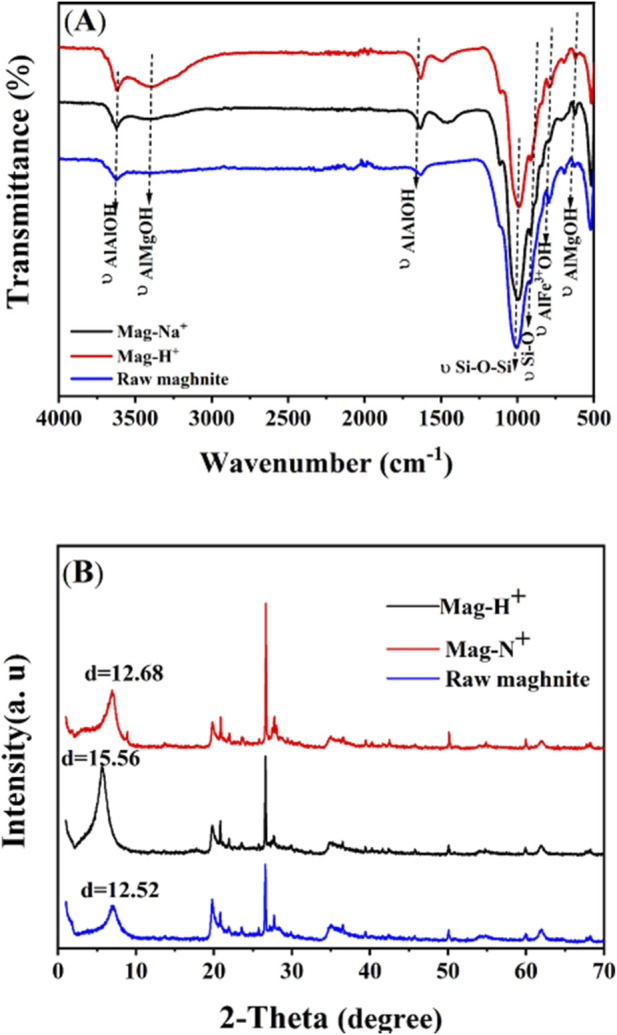
Physicochemical characterization of raw and modified maghnite (Mag-Na^+^ and Mag-H^+^). **(A)** FTIR spectra showing the effect of ion exchange on functional groups. **(B)** XRD patterns with calculated interlayer spacing (d) values, indicating structural changes after Na^+^ and H_3_O^+^ intercalation.

The X-ray diffraction (XRD) patterns (Figure 3B) clearly demonstrated structural differences between raw maghnite and its modified forms, maghnite-Na^+^ and maghnite-H^+^. Distinct diffraction peaks were observed at 2θ angles of 7.03° for raw maghnite, 5.91° for maghnite-Na^+^, and 5.61° for maghnite-H^+^. According to Bragg’s Law, these correspond to interlayer spacings of 12.52 Å, 12.68 Å, and 15.56 Å, respectively. The shift of diffraction peaks toward lower angles and the corresponding increase in basal spacing indicate interlayer expansion, which is characteristic of ion exchange and intercalation processes in layered clay minerals, as widely reported in previous studies (Mohan et al., 2022). The progressive increase in interlayer distance is attributed to the intercalation of Na^+^ and H_3_O^+^ ions into the sites previously occupied by Ca^2+^ and K^+^ cations, resulting in maghnite swelling. This ionic substitution modifies the crystalline structure and enhances crystallinity, which may significantly impact the functional properties of maghnite. Fourier Transform Infrared (FTIR) spectra (Figure 3A) revealed distinctive transmittance profiles for raw maghnite, maghnite-Na^+^, and maghnite-H^+^, highlighting the effect of ion exchange on the chemical environment of functional groups within the material. Broad absorption bands between 3,400 and 3,600 cm^-1^ were assigned to the stretching vibrations of structural hydroxyl groups (Al–Al–OH and Al–Mg–OH), which are typical of smectite-type clay minerals (Berkane et al., 2025; Yusuf, 2023). Broad absorption bands between 3,400 and 3,600 cm^-1^ were assigned to the stretching vibrations of Al–Al–OH and Al–Mg–OH groups. The band observed around 1,650 cm^-1^ is commonly associated with bending vibrations of adsorbed water and hydroxyl groups, consistent with previous FTIR studies of hydrated clay structures (Addadi et al., 2025). Characteristic deformation bands at 1,650 cm^-1^ (Al–Al–OH), 790 cm^-1^ (Al–Fe^3+^–OH), and 610 cm^-1^ (Al–Mg–OH) further confirmed the presence of these hydroxyl groups. Bands in the range of 980–1,000 cm^-1^ were attributed to Si–O stretching and Si–O–Si interactions, which represent the fundamental framework vibrations of silicate layers and are considered diagnostic of the tetrahedral structure of aluminosilicates (Vasconcelos et al., 2023), indicating that the structural differences among the samples are primarily due to the ionic modifications.

### 3.2 Reaction time and NBMP and poly (NBMP) yields

**FIGURE 4:**
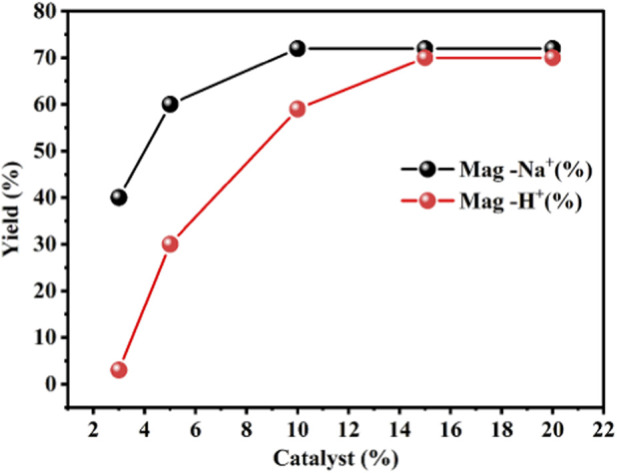
Effect of maghnite-H^+^ and maghnite-Na^+^ catalyst concentration on the yields of NBMP monomer and poly (NBMP) polymer.

A series of synthesis experiments were performed by keeping the reaction time constant (2 h for the monomer and 24 h for the polymer) and maintaining the temperature at 5 °C for the monomer and 0 °C for the polymer, while varying the catalyst amounts (3, 5, 10, 15, and 20 wt%; Table 1). During monomer synthesis, increasing the maghnite-H^+^ catalyst concentration from 3% to 5% increased the yield from 40% to 60%. Further increases to 10%, 15%, and 20% resulted in a maximum yield of 72%, indicating that catalyst concentrations above 10% do not significantly enhance NBMP yield. For NBMP polymerization, the polymer yield was only 5% with 3% maghnite-Na^+^, increasing to 30% with 5% catalyst and reaching 59%–70% with 10%–20% catalyst, respectively. These results suggest that 15% catalyst represents the optimal concentration for NBMP polymerization, whereas monomer synthesis can be effectively achieved with as low as 10% catalyst (Figure 4). The rapid reaction time required to obtain a 70% poly (NBMP) yield (24 h at 0 °C with 15% w/w catalyst relative to the monomer) contrasts sharply with conventional polymerization methods, which often require several hours to days. Moreover, scaling up the monomer concentration under these optimized conditions further increased poly (NBMP) yield while preserving both its quality and antimicrobial activity. Maghnite-H^+^ and maghnite-Na^+^ not only accelerate NBMP polymerization but also enhance overall polymer yield, making this catalytic approach a practical and efficient method for poly (NBMP) synthesis. It is worth noting that maghnite-H^+^ and maghnite-Na^+^ are solid, heterogeneous catalysts that can be easily separated from the reaction mixture after polymerization by simple filtration or centrifugation. This enables efficient recovery, cleaning, and reuse of the catalysts multiple times without significant loss of activity, reducing waste, lowering production costs, and promoting sustainable manufacturing. In addition to their catalytic function, these clay-based catalysts can be incorporated into the polymer matrix as reinforcing fillers. Their layered mineral structure enhances mechanical strength, thermal stability, and barrier properties, improving the durability and functional performance of the polymer without compromising purity or yield. This dual functionality as both reusable catalysts and reinforcing agents makes maghnite-H^+^ and maghnite-Na^+^ highly valuable for producing high-performance, environmentally friendly polymer materials.

### 3.3 Chemical bonding of NBMP and Poly (NBMP)

**FIGURE 5:**
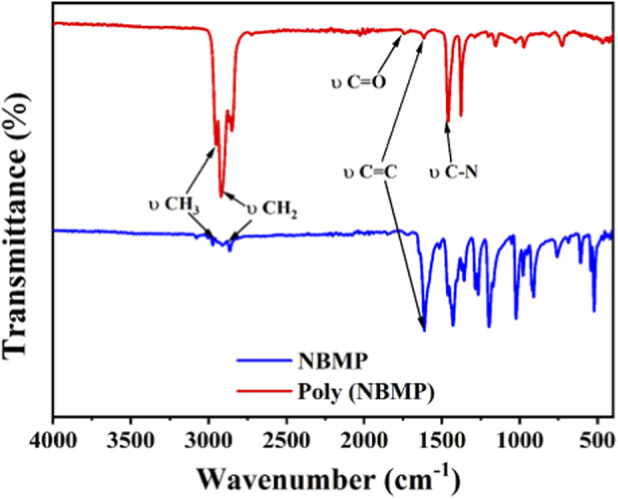
FTIR spectra of NBMP and poly (NBMP), highlighting key bond changes during polymerization. The disappearance of the C=C peak (1,613 cm^-1^) and the changes in CH_2_/CH_3_ stretching indicate successful formation of the polymer.

FTIR analysis of NBMP and poly (NBMP) revealed both similarities and key differences, providing insight into the chemical transformations during polymerization (Figure 5). Both monomer and polymer spectra showed prominent peaks at 2,871 cm^-1^ and 2,980 cm^-1^, corresponding to CH_2_ and CH_3_ stretching vibrations, indicating that the aliphatic backbone remained intact after polymerization. The carbonyl (C=O) stretching at 1722 cm^-1^, associated with the amide group, was also present in both spectra, suggesting retention of this functional group. Additionally, the C–N stretching peak at 1,431.69 cm^-1^, indicative of nitrogen linkages, was observed in both spectra, confirming the presence of nitrogen-containing moieties in the polymer backbone. Notable differences between the spectra confirmed successful polymerization. The C=C double bond peak at 1,613.46 cm^-1^, present in the monomer, disappeared in the polymer, indicating that the double bonds were converted to single bonds during chain formation. This transformation is a crucial step in poly (NBMP) synthesis, as it reflects the formation of covalent linkages between monomer units. Furthermore, the increased intensity of the CH_2_ and CH_3_ stretching peaks in the polymer spectrum suggests enhanced C–H vibrations due to the higher density of these groups along the polymer chain. The absence of the 522 cm^-1^ peak in the polymer indicates that certain bending vibrations characteristic of NBMP were lost, further supporting structural changes during polymerization. Overall, these spectral changes confirm the successful conversion of NBMP into poly (NBMP), highlighting the disappearance of double bonds and the establishment of new polymeric linkages.

### 3.4 NBMP and poly (NBMP) structure

The ^1^H NMR spectrum of NBMP in CDCl_3_ revealed characteristic signals confirming its molecular structure (Table 2). A singlet at δ 1.97 ppm was assigned to the methyl (-CH_3_) protons, while two singlets at 5.06 and 5.25 ppm (each integrating two protons) corresponded to the methylene (=CH_2_) protons of the vinyl groups, essential for polymerization. Multiplets at 3.60 ppm (8H) were attributed to N-CH_2_-CH_2_-N protons, confirming the integrity of the piperazine ring. Upon polymerization to poly (NBMP), several notable changes occurred. The methyl signal shifted to 0.76 ppm, indicating new electronic environments due to polymer chain formation. The disappearance of the vinyl methylene signals at 5.06 and 5.25 ppm confirmed the successful consumption of double bonds. A minor peak at 3.53 ppm suggested the presence of terminal hydroxyl groups, likely resulting from polymer solubility or interactions with residual solvents. The ^13^C NMR spectrum of NBMP showed signals at δ 20.53 ppm for the methyl group, 31.25–38.15 ppm for N-(CH_2_)_2_-N carbons, 116.17 ppm for the vinyl carbons (=CH_2_), 139.92 ppm for C=C, and 171.43 ppm for C=O, confirming the monomer’s functional groups (Table 3). In poly (NBMP), significant shifts were observed: the carbonyl signal moved from 171.40 ppm to 177.32 ppm, reflecting new hydrogen bonding and electronic interactions within the polymer matrix. A new peak at 30.05 ppm, assigned to quaternary carbons, indicated additional structural complexity introduced by polymerization. These NMR changes collectively confirm the successful transformation of NBMP into poly (NBMP) with preserved piperazine units and enhanced structural stability.

**TABLE 2:** ^1^H NMR chemical shifts of NBMP (monomer) and poly (NBMP).

Sample	Attribution	Chemical shift (δ ppm)
NBMP	–CH_3_ (a)	1.97
	= CH_2_ (b)	5.06 and 5.25
	–CH_2_– cyclic (c)	3.60
Poly(NBMP)	–CH_3_ (a)	0.76
	–CH_2_– cyclic (b)	1.49
	–CH_2_– aliphatic (c)	0.99–1.35
	–OH (d)	3.53

**TABLE 3:** ^13^C NMR chemical shifts of NBMP (monomer) and poly (NBMP).

Sample	Attribution	Chemical shift (δ ppm)
NBMP	–CH_3_ (a)	20.49
	–CH_2_– cyclic (b)	47.02
	= CH_2_ (c)	116.22
	–C=C (d)	139.92
	C=O (e)	171.40
Poly(NBMP)	–CH_3_ (a)	20.89
	–CH_2_– cyclic (b)	48.99
	–CH_2_– Aliphatic (c)	41.55
	C quaternary (d)	30.05
	C=O (e)	177.32

### 3.5 Poly (NBMP) morphological and thermal characteristics

**FIGURE 6:**
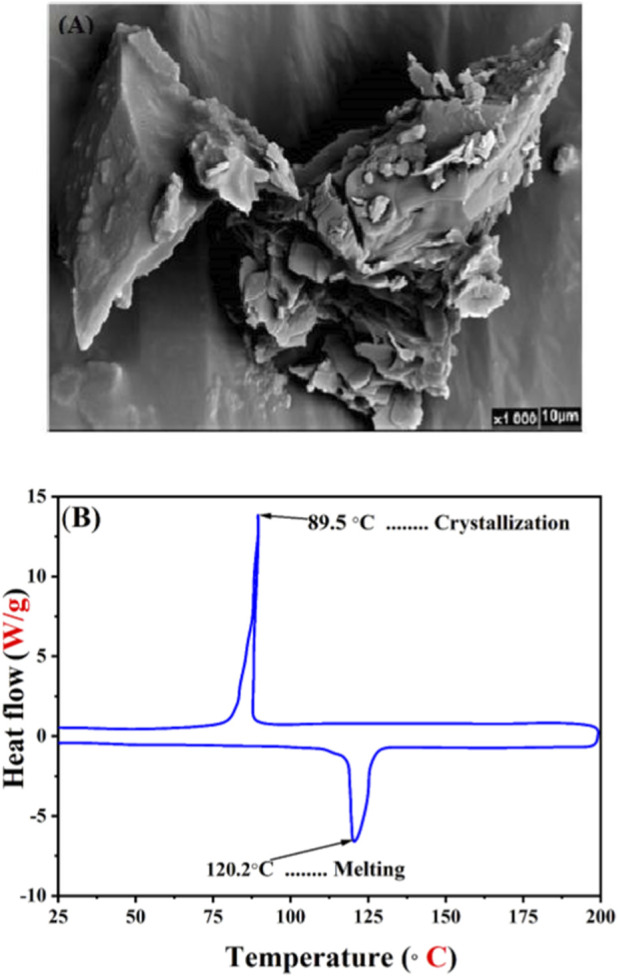
Poly (NBMP) characterization: **(A)** Representative SEM image showing well-organized crystalline aggregates; **(B)** DSC thermogram depicting thermal behavior with melting temperature (Tm), crystallization temperature (Tc), and associated enthalpies (ΔHf and ΔHc).

The surface morphology of the synthesized poly (NBMP) was examined using Scanning Electron Microscopy (SEM) (Figure 6A). The images revealed a well-organized structure with regular aggregates, indicative of the polymer’s crystalline nature and uniform microstructure. Differential Scanning Calorimetry (DSC) analysis provided insights into the thermal behavior of poly (NBMP) (Figure 6B). The polymer exhibited a melting temperature (Tm) of 120.23 °C and a fusion enthalpy (ΔHf) of 216.3 J/g, reflecting a robust crystalline structure that contributes to its thermal stability and mechanical strength. The crystallization temperature (Tc) was observed at 89.5 °C with a crystallization enthalpy (ΔHc) of 213.2 J/g, indicating effective crystallization behavior and a high degree of crystallinity. The close alignment between ΔHf and ΔHc values highlights the polymer’s reversible thermal behavior, which is advantageous for applications requiring resistance to temperature fluctuations. No distinct glass transition temperature (Tg) was observed at 65 °C in the DSC thermogram. This absence is consistent with the high crystallinity of poly (NBMP), where the proportion of amorphous regions is limited. In highly crystalline polymers, the restricted mobility of polymer chains within the crystalline domains reduces the visibility of Tg. Overall, the combination of high Tm, substantial ΔHf, and strong crystalline characteristics underscores poly (NBMP) as a thermally stable polymer with mechanical durability suitable for demanding applications.

### 3.6 Antimicrobial activity of Poly (NBMP)

**FIGURE 7:**
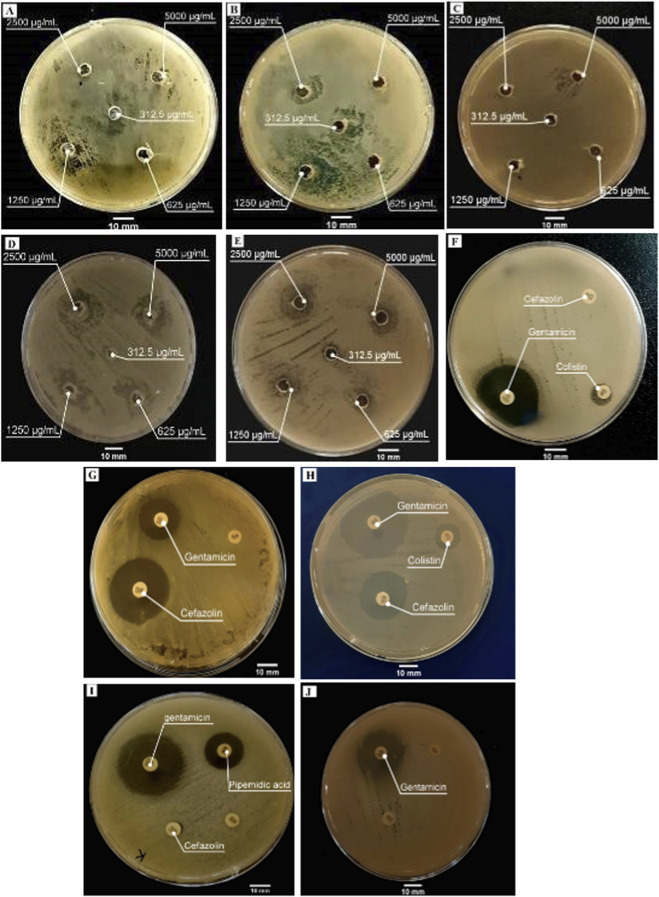
Antibacterial activity of poly (NBMP) at different concentrations (625, 1,250, 2,500, and 5,000 μg/mL) against *E. coli* (Gram-negative, **(A)**, *S. aureus* (Gram-positive, **(B)**, *P. aeruginosa* (Gram-negative, **(C)**, *K. pneumoniae* (Gram-negative, **(D)**, and *L. monocytogenes* (Gram-positive, **(E)**, compared with standard antibiotics against the same bacterial strains (**(F–J)**, respectively).

The antibacterial activity of poly (NBMP) was evaluated against Gram-positive and Gram-negative bacterial strains and compared with standard antibiotics (Tables 4, 5, and Figure 7). Gram-positive bacteria: Poly (NBMP) exhibited activity against *S. aureus* and *L. monocytogenes*. At 5,000 μg/mL, inhibition zones were 12.33 ± 0.92 mm for *S. aureus* and 21.40 ± 1.60 mm for *L. monocytogenes*. Interestingly, at a reduced concentration of 2,500 μg/mL, inhibition zones increased slightly to 13.40 ± 1.09 mm (*S. aureus*) and 23.87 ± 0.10 mm (*L. monocytogenes*), suggesting a concentration-dependent effect with optimal interaction at intermediate concentrations. For comparison, gentamicin (50 µg/disc) produced inhibition zones of 19.62 ± 1.43 mm and 25.43 ± 0.37 mm, respectively. Gram-negative bacteria: Poly (NBMP) also inhibited *E. coli*, *P. aeruginosa*, and *K. pneumoniae*. At 5,000 μg/mL, inhibition zones ranged from 10.80 ± 0.55 mm (*E. coli*) to 21.40 ± 1.60 mm (*K. pneumoniae*). Reducing the concentration to 2,500 μg/mL caused a marked decrease for *P. aeruginosa* (from 15.86 ± 0.97 to 6.00 ± 0.00 mm), indicating limited efficacy at lower doses. Gentamicin exhibited stronger activity against Gram-negative strains (28.08 ± 1.58 mm for *E. coli* and 34.00 ± 2.46 mm for *P. aeruginosa*), while cefazolin and colistin showed minimal or no inhibition for most strains. These results highlight that poly (NBMP) possesses concentration-dependent antibacterial activity, with variable efficacy across different bacterial species. Its activity is particularly notable against Gram-positive bacteria and some Gram-negative strains, suggesting potential as a novel antimicrobial agent. The antimicrobial mechanism of poly (NBMP) is likely related to its piperazine-based structure, which can disrupt bacterial cell membranes, interfere with DNA synthesis, and perturb essential cellular processes. Methacryloyl functional groups may enhance interactions with bacterial proteins or nucleic acids, further destabilizing cellular function, causing leakage of intracellular contents, and ultimately leading to cell death.

**TABLE 4:** Antibacterial activity of poly (NBMP) at different concentrations against Gram-positive and Gram-negative bacteria compared to standard antibiotics.

Compound	Conc.	Gram-positive bacteria		Gram-negative bacteria		
		*S.* *aureus*	*L.* *monocytogenes*	*E. coli*	*P. aeruginosa*	*K. pneumoniae*
poly (NBMP)	5,000 μg/mL	12.33 ± 0.92a	21.40 ± 1.60b	10.80 ± 0.55 c	15.86 ± 0.97d	21.40 ± 1.60b
	2,500 μg/mL	13.40 ± 1.09a	23.87 ± 0.10b	9.08 ± 0.18 c	6.00 ± 0.00d	23.87 ± 0.10b
	1,250 μg/mL	20.49 ± 0.91a	19.70 ± 7.44b	22.86 ± 3.48 c	6.00 ± 0.00d	19.70 ± 7.44b
	630 μg/mL	12.00 ± 0.42a	22.16 ± 1.69b	12.60 ± 1.49a	6.00 ± 0.00c	22.16 ± 1.69b
	312 μg/mL	20.43 ± 1.38c	11.65 ± 1.45b	17.19 ± 3.98 a	6.00 ± 0.00c	11.65 ± 1.45b
Gentamicin	50 µg/disc	19.62 ± 1.43a	25.43 ± 0.37b	28.08 ± 1.58 c	34.00 ± 2.46d	31.03 ± 1.02e
Cefazolin	20 µg/disc	28.93 ± 0.03a	0 ± 0.00b	0.00 ± 0.00b	25.53 ± 1.26c	7.92 ± 0.18d
Pipemidic	30 µg/disc	0 ± 0.00a	0 ± 0.00a	0.00 ± 0.00 a	0.00 ± 0.00a	18.19 ± 0.45b
Colistin	10 µg/disc	0 ± 0.00a	0 ± 0.00a	9.80 ± 0.04 b	13.30 ± 0.04b	0.00 ± 0.00a

Rows that do not share a common letter (a–e) differ significantly (p < 0.05).

**TABLE 5:** Predicted physicochemical, pharmacokinetic, and drug-likeness properties of the NBMP Monomer using SwissADME.

Predicted properties	Predicted value
Physical properties	
Formula	C_12_H_18_N_2_O_2_
Molecular weight	222.28 g/mol
Number of heavy atoms	16
Number of carbon atoms	0
Fraction of Csp^3^ carbons	0.50
Number of rotatable bonds	4
Number of hydrogen bond acceptors	2
Number of hydrogen bond donors	0
Molar refractivity	70.76
Topological polar surface area (TPSA)	40.62 Å^2^
Log Kp (skin permeation)	−7.00 cm/s
Drug-likeness	
Indicator (lipophilicity)	Value
Log P_0_/w (iLOGP)	2.47
Log P_0_/w (XLOGP3)	0.92
Log P_0_/w (WLOGP)	0.05
Log P_0_/w (MLOGP)	0.73
Log P_0_/w (SILICOS-IT)	1.20
Consensus log P_0_/w	1.08
Bioavailability score	0.55
Water solubility	
Property	Value
Log S (ESOL)	−1.53
Solubility (ESOL)	6.50 mg/mL; 0.0293 mol/L
Class	Very soluble
Log S (Ali)	−1.36
Solubility (Ali)	9.73 mg/mL; 0.0438 mol/L
Class	Very soluble
Log S (SILICOS-IT)	−1.23
Solubility (SILICOS-IT)	13.0 mg/mL; 0.0583 mol/L
Class	Soluble
Pharmacokinetic parameters	
Indicator	Value
Lipinski rule violations	Yes; 0 violations
Ghose rule violations	Yes
Veber rule violations	Yes
Egan rule violations	Yes
Muegge rule violations	Yes
PAINS alerts	0
Brenk alerts	1 alert: Michael acceptor 1
Lead-likeness	No; 1 violation: MW < 250
Synthetic accessibility	1.92

### 3.7 Drug-likeness and toxicity prediction of the NBMP monomer

**FIGURE 8:**
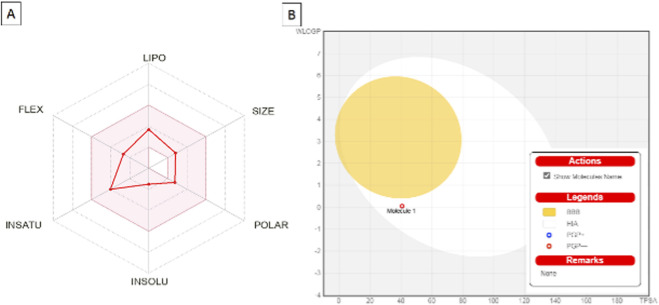
Bioavailability and BBB permeability analysis of the NBMP monomer: **(A)** The oral bioavailability radar (SwissADME) shows the ideal physicochemical range for optimal absorption and, NBMP characteristics (red line). **(B)** BOILED-Egg indicates low likelihood of transport by P-glycoprotein for NBMP (Molecule 1) and thus absence of absorption across the BBB.

**FIGURE 9:**
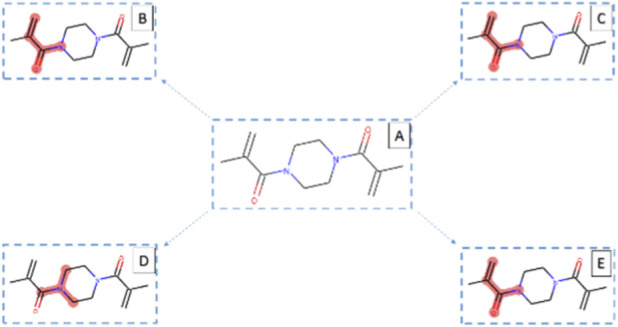
Toxicity prediction analysis of the NBMP monomer; **(A)** NBMP molecular structure; **(B)** Potential electrophilic agents; **(C)** Covalent binding interactions with proteins and **(D) (E)** Covalent binding interactions with DNA. The identified toxic substructures are (highlighted in red).

The physicochemical and pharmacokinetic properties of the NBMP monomer were evaluated (Table 5 and Figure 8) indicate that it is a promising drug candidate. With a molecular formula of C_12_H_18_N_2_O_2_ and a molecular weight of 222.28 g/mol, NBMP falls within the optimal range for drug development, favoring absorption and distribution. Typically, molecules with molecular weights ≤500 g/mol exhibit enhanced bioavailability, a key factor for therapeutic efficacy (Singh et al., 2022). The solubility profile of NBMP is also favorable. Its Log S (ESOL) value of −1.53 corresponds to “very soluble,” with a solubility of 6.50 mg/mL (0.0293 mol/L), indicating good gastrointestinal absorption and promising oral bioavailability (Savjani et al., 2012). However, the compound exhibits limited permeability to the blood-brain barrier (BBB), as suggested by a Log Kp (skin permeation) of −7.00 cm/s, which restricts its potential use in central nervous system therapies (Geldenhuys et al., 2015). From a pharmacokinetic perspective, NBMP displays several advantages: It is not a substrate for P-glycoprotein, reducing the risk of poor absorption and limited bioavailability (Daina et al., 2017). It does not inhibit key cytochrome P450 enzymes (CYP1A2, CYP2C19, CYP2C9, CYP2D6, CYP3A4), suggesting a low likelihood of drug-drug interactions, which is crucial for multi-drug therapies (Murray, 2006). Drug-likeness analysis showed that NBMP complies with multiple rules, including Lipinski, Ghose, Veber, Egan, and Muegge, without violations (Daina et al., 2017). Its consensus Log P_0_/w of 1.08 indicates balanced lipophilicity, supporting adequate absorption and distribution. Individual log P values obtained via different models—iLOGP: 2.47, XLOGP3: 0.92, WLOGP: 0.05, MLOGP: 0.73, SILICOS-IT: 1.20—further confirm a favorable profile for oral administration (Daina et al., 2017; Singh et al., 2022). Despite these promising characteristics, a lead similarity violation was noted due to its molecular weight being below 250 g/mol, which could be associated with lower potency and efficacy (Chuprina et al., 2010). Additionally, the synthetic accessibility score of 1.92 indicates potential challenges for large-scale synthesis, highlighting considerations for practical production, as complex synthetic routes can limit pharmaceutical development (Ertl and Schuffenhauer, 2009). Overall, NBMP demonstrates a favorable drug-likeness and pharmacokinetic profile, with good solubility, absorption potential, and low toxicity risk, although synthesis feasibility and CNS applicability remain limitations. Figure 9 illustrates these pharmacokinetic attributes. The oral bioavailability radar (left panel) shows that NBMP resides in a favorable zone for oral absorption. The BOILED-Egg plot (right panel) indicates that the compound is unlikely to cross the blood–brain barrier via P-glycoprotein, corroborating the earlier finding of limited CNS permeability, a critical consideration in CNS drug design (Geldenhuys et al., 2015). Toxicity assessment using the eMolTox web-based tool provided insights into the safety profile of NBMP. Despite its promising therapeutic properties, the tool identified potential toxic substructures, including risks associated with electrophilic agents and the formation of covalent bonds with DNA and proteins, which could interfere with essential biological functions (Yang et al., 2023). Although data-driven models predicted no significant toxic activity, these results suggest the need for caution and further in-depth investigations (Dahlin et al., 2015).

### 3.8 Molecular interaction assessment

**FIGURE 10:**
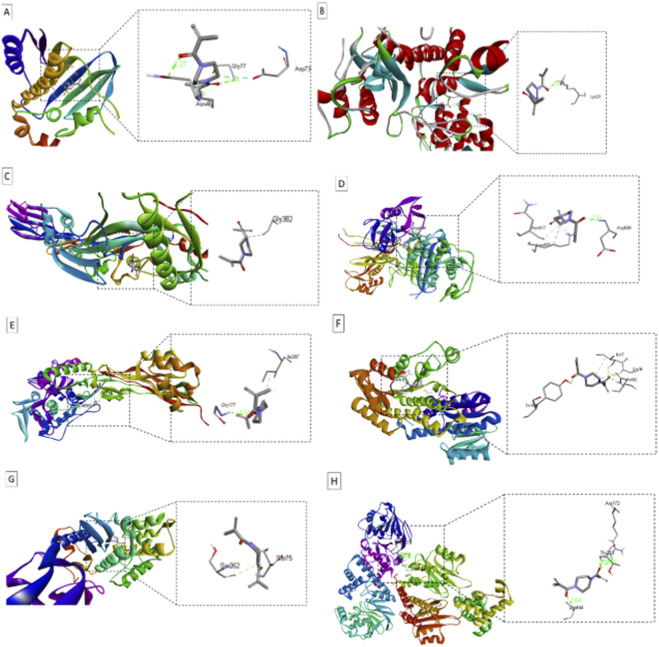
Surface views of protein-ligand complexes, active sites, and 3D bonds of NBMP with target proteins from *E. coli* (**(A)**: 1KZN), *S. aureus* (**(B,D,G)**: 3HUN, 5OJ0, 7KCY), *L. monocytogenes* (**(C)**: 4CDB), *K. pneumoniae* (**(F)**: 6T77), and *P. aeruginosa* (**(E,H)**: 6R3X, 8DQ1).

Molecular docking results (Table 6; Figure 10) demonstrated moderate interactions between NBMP and eight bacterial target proteins from both Gram-positive and Gram-negative species, including *E. coli* (1KZN), *S. aureus* (3HUN, 7KCY), *L. monocytogenes* (4CDB, 8DQ1), *K. pneumoniae* (5OJ0, 6T77), and *P. aeruginosa* (6R3X). Redocking experiments were performed to evaluate the reliability of the docking protocol, and the Vina RMSD values obtained for the top-ranked poses (Table 6) indicate consistent docking within the defined binding pockets. The binding affinities, expressed as estimated free energies of binding (kcal/mol), indicate the strength and stability of the ligand-protein complexes, which correlate with the potential antimicrobial activity of NBMP (Limongelli, 2020). The complex with the *K. pneumoniae* protein 6T77 exhibited a moderate binding affinity of −6.1 kcal/mol, involving residues Ile17, Gly18, Asn96, and Tyr151, suggesting a strong and stable interaction that may enhance the polymer’s antibacterial effectiveness (Zhang et al., 2021). Similarly, NBMP displayed significant binding to *E. coli* (1KZN, −6.2 kcal/mol) and *S. aureus* targets (3HUN and 7KCY, both −5.8 kcal/mol), with interactions involving residues such as Asn46, Gly77, Lys221, and Ser262. These residues contribute to binding stability through hydrogen bonds, hydrophobic contacts, and van der Waals forces, supporting diverse modes of molecular recognition (Patil et al., 2010). These findings suggest a potential binding ability of NBMP toward important bacterial proteins, providing valuable structural insights that may guide the design of novel antimicrobial agents.

**TABLE 6:** Binding affinities, interactions, and redocking validation of NBMP and co-crystallized ligands.

Protein (PDB ID) – Ligand complex	Estimated free energy of binding (kcal/mol)	Residues involved in bonded interaction	Co-crystallized ligand (formula)	Vina RMSD (Å) pose 1/2/3	Grid center coordinates (x, y, z)
1KZN – NBMP	−6.2	Asn 46, Gly 77	Clorobiocin (C_27_H_26_O_11_)	0.00/2.567/1.03	19.150, 30.393, 34.745
3HUN – NBMP	−5.8	Lys 221	Ampicillin (C_16_H_19_N_3_O_4_S)	0.00/2.16/1.57	−33.648, 13.630, −10.162
4CDB – NBMP	−5.4	Tyr 256	Tris (hydroxymethyl) aminomethane (C_4_H_11_NO_3_)	0.00/2.16/1.57	−11.691, −8.144, −14.361
5OJ0 – NBMP	−5.8	Asp 698	Cefepime (C_19_H_24_N_6_O_5_S_2_)	0.00/1.09/2.95	31.712, −15.361, 52.877
6R3X – NBMP	−5.9	Gly 177	Piperacillin (C_23_H_33_N_5_O_7_S_2_)	0.00/1.86/2.98	7.114, 40.689, 15.918
6T77 – NBMP	−6.1	Ile 17, Gly 18, Asn 96, Tyr 151	NADP (C_21_H_28_N_7_O_17_P_3_)	0.00/2.53/2.17	−12.659, −1.511, −13.323
7KCY – NBMP	−5.8	Ser 262	Cefoxitin (C_16_H_17_N_3_O_7_S_2_)	0.00/2.57/2.19	−6.960, 17.986, 15.628
8DQ1 – NBMP	−5.6	Ser 144, Arg 172, Thr 213	4-(3-bromophenoxy)-N-[(3S)-2-oxothiolan-3-yl]butanamide (C_14_H_16_BrNO_3_S)	0.00/2.26/2.17	130.594, 141.381, 163.014

### 3.9 Molecular dynamic simulations

**FIGURE 11:**
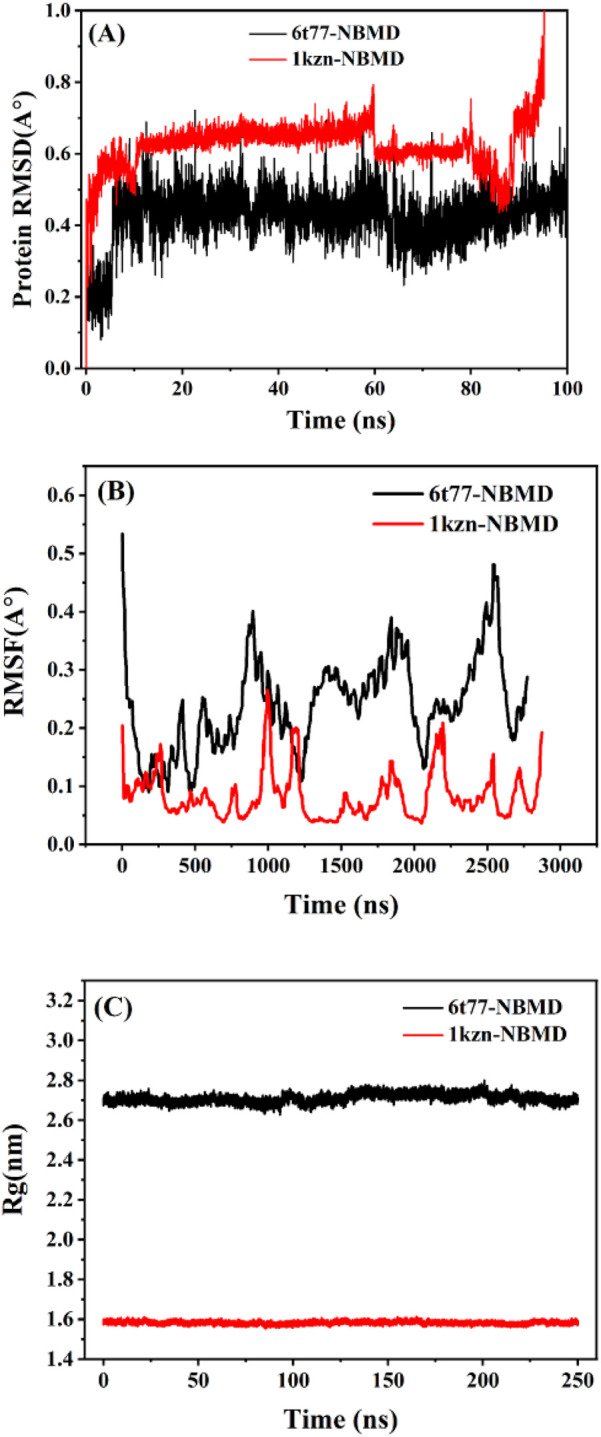
The molecular dynamics simulation analyses showing **(A)** RMSD (nm), **(B)** RMSF (nm), and **(C)** radius of gyration (Rg, nm) values of *E. coli* protein 1KZN and *K. pneumoniae* protein 6T77 complexed with NBMP.

Molecular dynamics (MD) simulations were performed to further investigate the stability of NBMP bound to bacterial proteins from *E. coli* (1KZN) and *K. pneumoniae* (6T77), following the docking studies. The top-performing protein–ligand complexes identified from docking were selected to assess the dynamic behavior of the interactions over time. Key parameters, including root mean square deviation (RMSD), root mean square fluctuation (RMSF), and radius of gyration (Rg), were analyzed to evaluate structural stability and compactness (Figure 11).

RMSD analysis measures the deviation of the protein backbone atoms relative to their initial positions, providing insight into structural stability. As illustrated in Figure 11A, both 1KZN–poly (NBMP) and 6T77–poly (NBMP) complexes reached equilibrium after an initial adjustment period of approximately 5.9 ns. The average RMSD values were 0.43 Å for 1KZN and 0.19 Å for 6T77, with the lower RMSD for 6T77 indicating particularly stable interactions. These findings suggest that NBMP forms robust and stable complexes with these proteins, which is critical for potential antibacterial activity. Literature indicates that stable RMSD profiles are indicative of reliable ligand-protein binding in drug design (Limongelli, 2020; Patil et al., 2010).

RMSF analysis provided a residue-level perspective of flexibility within the complexes, reflecting fluctuations of individual amino acids during the simulation (Figure 11B). Both complexes exhibited low RMSF values, ranging from 0.08 Å to 0.48 Å, demonstrating minimal flexibility and high structural stability around the binding sites. This indicates that NBMP maintains consistent interactions with key amino acid residues of *E. coli* and *K. pneumoniae* proteins, resisting conformational changes that could weaken binding (Sipahi, 2008; Zhang et al., 2021).

Finally, the radius of gyration (Rg) values, representing the overall compactness of the protein-ligand complexes, remained stable between 0.04 and 0.07 nm throughout the simulations (Figure 11C). The consistent Rg values indicate that the complexes retained their three-dimensional structure without significant unfolding or distortion, which is essential for preserving protein function and maintaining effective ligand binding. These results collectively confirm the strong binding affinity and structural integrity of NBMP complexes with *E. coli and K. pneumoniae* target proteins, supporting its potential as an effective antibacterial agent.

## 4 Conclusion

In this study, poly (NBMP) was efficiently synthesized via catalytic polymerization using green clay-based catalysts, maghnite-H^+^ and maghnite-Na^+^. This eco-friendly method reduced reaction time and improved yields. These biocompatible, low-toxicity catalysts are easily recoverable and reusable, making the process sustainable, cost-effective, and environmentally friendly while ensuring high product purity. The monomer (NBMP) yield increased significantly from 40% to 72% using 0–10 wt% Maghnite-H^+^ at 5 °C over 2 h, while the polymer yield rose from 5% to 70% with 0–15 wt% catalyst at 0 °C over 24 h, indicating the effective catalytic activity of both Maghnite-H^+^ and Maghnite-Na^+^. Structural characterization via FTIR, ^1^H NMR, ^13^C NMR, DSC, and SEM confirmed the formation of the desired polymer and the presence of functional groups critical for bioactivity. Poly (NBMP) exhibited potent antimicrobial activity, with inhibition zones observed at 32 μg/mL against *Staphylococcus aureus* and 16 μg/mL against *Escherichia coli*, indicating broad-spectrum potential. In silico drug-likeness evaluation and molecular docking revealed strong binding affinities, especially with the *Klebsiella pneumoniae* FabG protein (PDB ID: 6T77), showing a binding energy of −6.1 kcal/mol and key interactions with Ile17, Gly18, Asn96, and Tyr151. Molecular dynamics simulations further validated the stability of the NBMP complexes with *E. coli* DNA gyrase (PDB ID: 1KZN) and *K. pneumoniae* FabG (6T77), with RMSD values of 0.43 nm and 0.19 nm, respectively. Low RMSF (0.08–0.48 nm) and compact Rg values (0.04–0.07 nm) indicated minimal structural fluctuations and stable complex formation. The poly (NBMP) showed strong antimicrobial potential and favorable drug-likeness, making it a promising candidate for further development as a novel antimicrobial agent. Future studies should focus on further physicochemical characterization, detailed *in vivo* evaluations, and optimization of polymer formulations to enhance their biomedical applicability and therapeutic performance.

## Data Availability

The raw data supporting the conclusions of this article will be made available by the authors, without undue reservation.
